# Anemia among Primary School Children in Eastern Ethiopia

**DOI:** 10.1371/journal.pone.0123615

**Published:** 2015-04-22

**Authors:** Firehiwot Mesfin, Yemane Berhane, Alemayehu Worku

**Affiliations:** 1 College of Health and Medical Sciences, Haramaya University, Harar, Ethiopia; 2 Addis Continental Institute of Public Health, Addis Ababa, Ethiopia; 3 School of Public Health, Addis Ababa University, Addis Ababa, Ethiopia; University of São Paulo, BRAZIL

## Abstract

**Background:**

Anemia during childhood impairs physical growth, cognitive development and school performance. Identifying the causes of anemia in specific contexts can help efforts to prevent negative consequences of anemia among children. The objective of this study was to assess prevalence and identify correlates of anemia among school children in Eastern Ethiopia.

**Methods:**

A cross sectional study was conducted from January 2012 to February 2012 in Kersa, Eastern Ethiopia. The study included randomly selected primary school students. Hemoglobin concentration was measured using a Hemocue haemoglobinometer. A child was identified as anemic if the hemoglobin concentration was <11.5 g/dl for children (5–11 yrs) and < 12 g/dl for child older than 12 years age. Poisson regression model with robust variance was used to calculate prevalence ratios.

**Result:**

The overall prevalence of anemia was 27.1% (95% CI: 24.98, 29.14): 13.8% had mild, 10.8% moderate, and 2.3% severe anemia. Children with in the age group of 5-9 years (APR, 1.083; 95% CI, 1.044- 1.124) were at higher risk for anemia. Paternal education (Illiterate, 1.109; 1.044 - 1.178) was positively associated with anemia. Children who had irregular legume consumption (APR, 1.069; 95% CI, 1.022 -1.118) were at higher risk for anemia.

**Conclusion:**

About a quarter of school children suffer from anemia and their educational potential is likely to be affected especially for those with moderate and severe anemia. Child age, irregular legume consumption, and low paternal schooling were associated with anemia. Intervention programmes aimed to reduce anemia among school children are crucial to ensure proper growth and development of children.

## Introduction

World Health Organization estimated that about 40% of the world’s population (more than 2 billion people) suffers from anemia. It is pervasive among schoolchildren. More than half of whom are estimated to suffer from iron deficiency anemia [1]. Studies from Ethiopia also showed that the prevalence of anemia among school children ranges from 5.8%–37.6%, which indicated that anemia, is a public health problem [2–8].

Anemia impairs the immune mechanisms, and causes increased morbidity which may lead to fatigue, low productivity, and a general sense of feeling unwell [9]. In school children it impairs physical growth, cognitive development and school performance [10].

Anemia is the result of a wide variety of causes that often coexist together with other micronutrient deficiencies. Iron deficiency is the most significant contributor to anemia [3, 11]. socioeconomic factors such as maternal education, gender norms, and low income [12]; infectious diseases such as malaria, worm infestation and schistosomiasis [13, 14, 15, 16] and deficiencies of other essential micronutrients such as vitamin A, folic acid and vitamin B12 [17].

Although anemia is a public health problem in Ethiopia, data on anemia among school children are scarce. The revised National Nutrition Program of Ethiopia (2013) clearly indicated the need to conduct a comprehensive and routine nutritional assessment at schools. Therefore, the objective of this study was to investigate the prevalence and correlates of anemia among school children in Eastern Ethiopia.

## Materials and Methods

This cross sectional study was conducted among school children aged 5–14 years in 12 public primary schools of Kersa district, East Hararge Zone, Oromia regional state, Eastern Ethiopia. Kersa District is one of the districts in the zone and comprises of 38 kebeles (the smallest administrative units) in Ethiopia. KDS-HRC comprises 10256 households and a total population of 48192. The DSS has three climatic zones, low land, high land, and midland and stratified as ten rural kebeles and two semi—urban areas. In all study sites there are health extension workers or community health workers who provide basic primary health care services. Moreover, there are only three Health Centers within the geographic coverage of the DSS but there is no hospital. The health care coverage of the district was 80% in 2010. According to the Office of Education in Kersa district there were 18 elementary, 2 secondary, 1 preparatory, and 2 religious schools in the study Kebeles. The livelihood of the study population is mainly dependant on subsistence agriculture. But small trade, government employment and daily work are also means of living. The District is known by its scarce crop, roots and other vegetables production. Subsistent crops are often planted during the wet season (June-September) and harvested during the dry season (October—February). There are no profound cultural taboos related to eating habits. The survey was conducted during January—February 2012.

The study population was student-parent pair, students were identified in schools and their parents contacted through the demographic surveillance system operating around the schools selected for the study. The calculated sample size was 1832 assuming anemia prevalence of 21% at 95% confidence level and a margin of error of 2 [16]. Fifteen percent non-response rate was added to the sample size as a contingency.

Students were selected randomly proportional to the student size of the study schools. In each school sampling frame was prepared from the student roster in each grade and students were selected from each grade by simple random sampling method. All schools involved in this study were located within the geographic limit of the Demographic and Health surveillance (KDH-HRC) site which is managed by Haramaya University (the host of the principal investigator). Thus, parents were contacted through the surveillance field workers.

Data were collected using a pre-tested structured questionnaire translated into the local language (*Afan Oromo*) by trained and experienced data collectors who were fluent in the local language. The data collection tool was prepared based on the national survey questionnaire [4]. Respondents of the questionnaire were parents/caregivers of the children identified in the study schools. After students were selected randomly in the schools their household number was traced in the KDH-HRC database. Then, data collectors visited the children’s house to administer the questionnaire to one of the parents/care takers.

Clinical assessment of Vitamin A deficiency (Bitot’s spots, corneal ulceration, and corneal scarring) was performed through physical examination of the child by trained nurses. Information about night-blindness was collected by asking the mothers regarding the status of the child in seeing at dusk or in dim light. In addition mothers/care takers were asked about whether the child was sick and treated during the previous fifteen days prior to the survey.

Hemoglobin determination was done by laboratory technicians for the selected students in the school compound. The haemoglobin concentration of each student was measured by taking a finger-prick blood sample using a Hemocue haemoglobinometer (Hemocue, angelholm, Sweden). A prick was made on the tip of the middle finger after the site was cleaned with disinfectant. The first drop of blood was cleaned off and the second drop (0.05ml) was collected to fill the microcuvette which is then placed in the cuvette holder of the device (HemoCue Hb 301^+^) for measuring hemoglobin concentration. The displayed hemoglobin value was then recorded. The technique is recommended by WHO for use in field surveys [18, 19]. Data collection was supervised daily by trained field supervisors and the principal researcher.

Hemoglobin level was divided into four for two age categories. For children 5 to 11 year; ≥11.5 g/dl normal, 11.0–11.4 g/dl mild anemia, 8.0–10.9 moderate, and < 8.0 g/dl severe anemia. For children 12 to 14 year; ≥12 g/dl normal, 11.0–11.9 g/dl mild anemia, 8.0–10.9 moderate, and < 8.0 g/dl severe anemia [20, 21]. Hemoglobin concentrations were corrected for altitude as proposed by the WHO [21].

Based on the adapted conceptual model from previous studies [11, 12, 13] the independent variables included in the study were the child’s age, sex, morbidity status, clinical sign and symptoms of vitamin A deficiency, shoe wearing practice, the number of children in the household, educational status and occupation of parents, wealth index, age of the mother, dietary habit, morbidity status, water source and waste disposal system of the household.

Principal component analysis (PCA) was used to derive a wealth index from information on ownership of the household assets. Principal Components with eigenvalues greater than one were retained to construct wealth index values and then categorized into three relative measures of socio economic status of households as low, medium and high.

Structured food frequency questionnaire (FFQ) modified from the Helen Keller International FFQ that was used previously in Ethiopia [22, 23, 24], was used to obtain qualitative information about the usual food consumption patterns with an aim to assess the frequency with which certain food items or groups are consumed during a specific time period. Food frequency questionnaire that contains 25 items that are commonly consumed in the study area were grouped into seven as cereals, legumes, meat, egg, vegetables, fruits, and dairy products. The question, “How often do you usually eat/drink…” was followed by a list of food items, including “Fruits” and “Vegetables”. The options were: “Never”, “once per month”, “Once a week”, “2–4 days a week”, and “every day. The food frequency questionnaire was pretested in the field before the actual data collection.

Data were double entered using EpiData 3.1 software by trained data clerks. Bivariate and multivariate analyses were performed in the Stata software, version 11, where Poisson regression with robust variance was used to calculate prevalence ratios [25, 26]. Descriptive statistics, (frequency counts and percentages) were used to summarize categorical variables while mean and standard deviations were used to present continuous variables. The adapted conceptual model for risk factors of anemia among school-age children from previous studies [11, 12, 13] was used to select the variables in the regression model.

The multivariate model followed a hierarchical approach from distal to proximal determinants on three levels: [1] socio-economic factors; [2] environmental factors; [3] reported morbidity and dietary intake [27].

In this model, it was assumed that the outcome varies according to risk factors; although some are not directly responsible for the occurrence of the outcome, they potentiate the effect of other determinants. The demographic variables (sex and age), although they interfere with the outcome, are not influenced by other factors, but can be determinants over the rest tested at the beginning of modeling. Socioeconomic level exerts influence over environmental and nutritional factors, since it establishes exposure conditions. For this reason, socioeconomic factors were analyzed at the first level, together with demographic variables. At the second level were environmental factors, at the third level were dietary habit and reported morbidity status.

Variables were progressively introduced in to the model, according to the hierarchical level. Variables reaching statistical significance and those with p<0.20 in the first level were kept in the model and included in the adjustment of the next level. This same procedure was employed for the following level. Variables with a p-value<0.05 in the adjusted analysis were maintained in the final regression model.

Ethical clearance was obtained from the College of Health Science of Haramaya University. Capillary blood collection was performed after obtaining a signed written informed consent from parents and an oral assent from the children. Privacy and confidentiality were maintained at each step of the study process.

## Results

A total of 1755 school children (750 girls and 1005 boys) aged 5–14 years from twelve primary schools were included in the study. The mean (±SD) age was 10.7yrs (SD ±2.1) and 43.6% of the families had more than 5 children. The average age of the mothers was 36.2 with standard deviations of ±7.7 and the majority of them were illiterate (Table 1).

**Table 1:** Socio-demographic characteristics of primary school children and their parents, Kersa district, Eastern Ethiopia, 2012 (n = 1755).

Variable		Number	Percent
***Age***	5–9	539	30.7
	10–14	1216	69.3
***Child sex***	Male	1005	57.3
	Female	750	42.7
***Residence***	Semi urban	316	18.0
	Rural	1439	82.0
***Father education***	Illiterate/read-write	1087	68
	primary	383	22
	Secondary and above	130	7
***Father occupation***	Farmer	1396	85.2
	Daily laborer	118	7.2
	Government employee	124	7.6
***Maternal age***	24–34	915	57.9
	35–44	337	21.3
	> = 45	328	20.8
***Mother’s education***	Illiterate/read-write	1233	78
	Literate	347	22
***Mother’s occupation***	Housewife	1477	93.5
	Government employee	103	6.5
**Number of siblings**	< = 3	402	22.9
	4–5	588	33.5
	>5	765	43.6
***Wealth status***	Low	589	33.4
	Middle	588	33.3
	High	588	33.3

**Fig 1:**
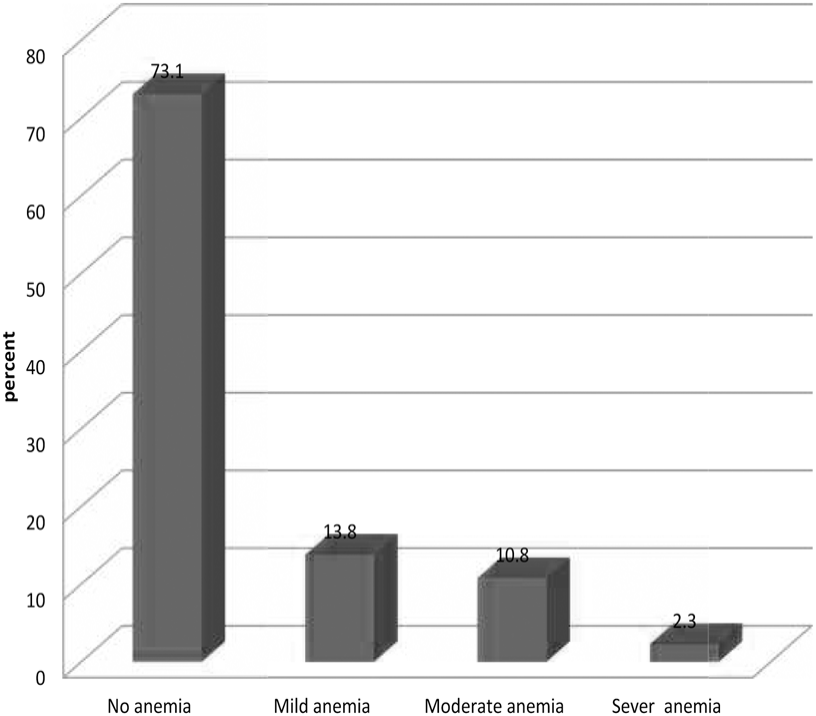
Prevalence of severity of anemia in Kersa primary school children. Classification of anemia was done based on WHO criteria into four as hemoglobin concentration ≥11.5 g/dl normal, 11.0–11.4 g/dl mild, 8.0–10.9 moderate, and < 8.0 g/dl severe anemia. for children age < 12 y and ≥12 g/dl normal, 11.0–11.9 g/dl mild, 8.0–10.9 moderate, and < 8.0 g/dl severe anemia for age > 12 y. The percentage of anemia appears in the top of the bar.

Prevalence of anemia among primary school children was 27.1% (95% CI; 24.98, 29.14): 13.8% were mildly anemic, 10.8% were moderately anemic, and 2.3% were severely anemic (Fig 1). The mean (±SD) hemoglobin level was 12.6 gm/dl (± SD 1.8). The prevalence of anemia among the age group 5–9 years was 188 (34.9%) and 287 (23.6%) among the age group 10–14 years old children. No statistically significant difference in the prevalence of anemia was observed between male and female children (27.3% and 26.8%). Anemia was prevalent among children from less educated mothers (29%) and fathers (31%). Similarly, children who had irregular legumes consumption (34.7%) were anemic.

In the unadjusted regression analysis, the following independent variables were associated with anemia: age of child, parental occupation, number of children in the house hold, paternal education, and consumption of fruit, legumes, yellow vegetables, meat and poultry, environmental characteristics (water source and waste disposal), illness in the last two weeks and history of night blindness.

To estimate the relative contribution of each risk factor, adjusted prevalence ratios (aPR) was derived from multiple Poisson regression models with anemia as the outcome. Children with in the age group of 5–9 years (APR = 1.083 95% CI: 1.044–1.124) were at higher risk for anemia. Similarly, children born from a low paternal education (APR = 1.109; 95% CI: 1.044–1.178) and who had an irregular legume consumption (APR = 1.069; 95% CI: 1.022–1.118) were at higher risk for anemia (Table 2).

**Table 2:** Hierarchical Poisson regression for anemia and independent variables among school children aged five-to fourteen years in Kersa district, Eastern Ethiopia, 2012 (n = 1755).

Variable		Anemia		Unadjusted PR *	Adjusted PR†
		Yes	No	(95% CI)	(95% CI)
		no (%)	no (%)		
***Age***	5–9	188 (34.9)	351 (65.1)	1.09 (1.05–1.13)	1.08(1.04–1.12)
	10–14	287 (23.6)	929 (76.4)	1	1
***Sex***	Male	274 (27.3)	731 (72.7)	1	1
	Female	201 (26.8)	549 (73.2)	0.99 (0.96–1.039)	0.99 (0.96–1.02)
**1** ^**st**^ **level**					
***Father education***	Illiterate/read-write	337 (31.0)	750 (69.0)	1.199 (1.14–1.26)	1.109(1.04–1.18)
	Primary	91 (23.8)	292 (76.2)	1.13 (1.070–1.19)	1.07(1.01–1.14)
	Secondary and above	12 (9.2)	118 (90.8)	1	1
***Mother’s education***	Illiterate/read-write	358 (29.0)	875 (71.0)	1.05 (1.01–1.09)	1.01 (0.95–1.05)
	Literate	78 (22.5)	269 (77.5)	1	1
***Father occupation***	Farmer	411 (29.4)	987 (70.6)	1.18 (1.12–1.24)	1.07(0.98–1.17)
	Daily laborer	27 (22.9)	112 (90.3)	1.12 (1.04–1.21)	1.04 (0.93–1.17)
	Government employee	12 (9.7)	91 (77.1)	1	1
***Mother’s occupation***	Housewife	416 (28.2%)	1061 (71.8)	1.07(1.00–1.15)	0.97 (0.89–1.05)
	Government employee	20 (19.4)	83 (80.6)	1	1
***Wealth status***	Low	155 (26.3)	434 (73.7)	0.99 (0.96–1.03)	0.99 (0.96–1.04)
	Middle	184 (31.3)	404 (68.7)	1.03 (0.97–1.09)	1.02 (0.96–1.08)
	High	159 (27.0)	431 (73.3)	1	1
**2** ^**nd**^ **Level**					
***Number of siblings***	<5	242 (24.4)	748 (75.6)	1	1
	> = 5	233 (30.5)	532 (69.5)	1.05 (1.01–1.08)	1.03 (0.99–1.07)
***Water source of HH***	improved	313 (23.8)	1004 (76.2)	1	1
	unimproved	112 (37)	276 (63)	1.11 (1.07–1.15)	1.03 (0.98–1.07)
***Waste disposal system***	Proper	89 (21.9)	318 (78.1)	1	1
	Improper	377 (28.6)	399 (71.4)	1.06 (1.02–1.09)	0.99 (0.95–1.04)
***Toilet***	No	156 (29.5)	372 (70.5)	0.97 (0.94–1.01)	1.02 (0.98–1.06)
	Yes	319 (26%)	908 (74.0)	1	1
**3** ^**rd**^ **Level**					
***fruit consumption***	No	475 (27.4)	1279 (72.6)	1.14 (1.09–1.19)	1.02(0.96–1.08)
	Yes	33 (12.8)	224 (87.2)	1	1
***legumes consumption***	No	205 (34.7)	385 (65.3)	1.12(1.08–1.16)	1.07(1.02–1.12)
	Yes	303 (21.6)	1098 (78.4)	1	1
***Yellow vegetables consumption***	No	267 (32)	568 (68.0)	1.09 (1.05–1.12)	1.01(0.97–1.05)
	Yes	241 (20.8)	915 (79.2)	1	1
***Meat and poultry consumption***	No	317 (30.5)	721 (69.5)	1.08 (1.05–1.12)	1.02(0.98–1.06)
	yes	191 (20)	762 (80.0)	1	1
***Child illness in the last 2 wks***	Yes	418 (26.2)	1175 (73.8)	1	1
	No	57 (35.4)	104 (64.6)	1.07 (1.01–1.13)	1.03(0.97–1.09)
**Night blindness**	No	412 (26)	1171 (74)	1	1
	Yes	63 (36.6)	109 (63.4)	1.08(1.03–1.14)	1.04 (0.98–1.09)

The percentage (%) shown in the table of yes column represents the frequency of cases exposed in each risk factors

** P< 0.001 significance level

* P< 0.005

PR (Prevalence Ratio)

## Discussion

The Government of Ethiopia has made the school children and adolescent health as National nutrition program (NNP) Package since 2013 [28]. Since then the government has put in place programmes and initiatives to ensure adolescents’ access to micronutrient services and to provide comprehensive and routine nutritional assessment and counseling services for adolescents at community, school and health facility. In addition to that the Ministry of Education also has a National School Health and Nutrition Strategy (SHN) to enable improved access to better health and nutrition services for school-age children through government and non-government schools [29]. With these strategies in place, findings obtained from the present study will provide the sectors with supportive information to implement the micronutrient interventions to prevent nutritional deficiencies in this outlook.

This study revealed that the overall prevalence of anemia as 27.1% in primary school children. It was associated with child’s age, paternal education and irregular consumption of legumes. The prevalence observed in this study showed that anemia among primary school children is a moderate public health problem as classified by WHO [20]. The level of anemia observed in this study was higher than the findings of similar studies conducted in Ethiopia [2–7]. However, it was lower as compared with study conducted in Jimma Town, Southwest Ethiopia (37.6%) [8] and in neighboring African countries, Tanzania 79.6%, Nigeria 82.6%, Uganda 47% [13, 14, 30, 31], and Asian countries India 53% [12] and Pakistan 59% [32]. These differences in the prevalence of anemia may be due to difference in the study area, sample size, the diet consumed and other associated factors.

In the present study, younger children (5–9 years) had higher prevalence of anemia (34.9.0%) than those 10–14 years old (23.6%). This finding is in agreement with study finding from Jimma which indicated that the prevalence of anemia among the age group of 6–11 years was 118(40.5%), while it was 34(30.1%) among the age group of 12–14 years [8]. However this result is in contrast with previous studies report in which anemia was higher among adolescents [33]. The high anemia prevalence observed among younger children could be explained by the high level of anemia (44%) known to exist among pre-school children (<5 years) in Ethiopia [34]. Children are possibly entering school with untreated anemia. This may indicate the low coverage of nutrition interventions to address anemia aimed to vulnerable preschool children.

In the current study maternal education did not show significant association with anemia among school children in contrary to the findings reported by several studies [35–37]. This could be due to the homogeneity of the respondents' educational status, as more than 75% of mothers had no formal education in this study. However, paternal education showed significant association with anemia among all of the factors comprising parent’s socio demographic characteristics that have been evaluated.

This finding is in agreement with studies identified paternal education as a stronger determinant of child stunting than maternal education in Indonesia and Bangladesh [38]. Expectedly, as the level of education of the fathers increases, so do his finances and his contribution to the total family income and better nutritional status. In addition, fathers who are educated are more likely to make decisions that will improve nutrition and health of their children. An educated father is likely to send all his children to school, thereby breaking the chain of ignorance and contributes independently to long-run child nutrition [39].

The association of irregular consumption of legumes with higher risk of anemia in this study could be explained by low intake of phytoferritin. Phytoferritin iron from legume represents the majority of the total iron in seeds, indicating that the main function of phytoferritin is iron storage [40, 41]. Recent studies have shown that iron absorption from phytoferritin in the body is better as compared to other non heme iron, because of the iron inside phytoferritin is protected by a protein shell that protects it from interactions with other dietary elements such as phytate [42].

Understanding the mechanism for iron absorption from legumes and promoting their consumption could help solve iron deficiency anemia by providing an affordable and readily available alternative source of iron in the diet. There is now an initiative to use phytoferritin as iron supplement for treatment of iron deficiency anemia [43].

Limitation of this study is that the serum ferritin and serum level of key micronutrients (Vit A and Vit B12) were not measured and the presence of parasitic infections like helminthes were not investigated which could still be more widely prevalent. Such data may provide useful information to explain multi factorial causes of anemia in the population studied. However, the clinical signs and symptoms of vitamin A deficiency and shoe wearing practice of the children did not show any significant association with anemia.

The other limitation was the food frequency questionnaire, which is retrospective method that relies upon the respondent’s memory; less sensitive to measures of absolute intake for specific nutrients; arbitrary groupings of foods may not correspond to the perception of the respondent; exclusion of foods popular to ethnic minority groups that are significant contributors of nutrients will skew the data. To minimize this problem the list of food items was developed based on questionnaire that have been used in previous national studies and an interview of the key informants who are from the study area and who knew the culture and language on the types of foods commonly consumed.

The representativeness of the sample studied and the implementation of tested and validated methods and tools represent strength of the present study. To minimize misclassification, we used the WHO criteria for determining the presence of anemia, based on hemoglobin cutoff values for age and with an additional epidemiological criterion for assessing the severity and magnitude of the problem.

In conclusion, over a quarter of school children suffer from anemia and it is associated with child age, low literacy status of father and irregular consumption of legumes. The observed level of anemia is likely to interfere with the intellectual potential of the children. Appropriate school based interventions can help improve the situation.
